# Sperm Motility, Oxidative Status, and Mitochondrial Activity: Exploring Correlation in Different Species

**DOI:** 10.3390/antiox10071131

**Published:** 2021-07-16

**Authors:** Alessandra Gallo, Maria Consiglia Esposito, Elisabetta Tosti, Raffaele Boni

**Affiliations:** 1 Department of Biology and Evolution of Marine Organisms, Stazione Zoologica Anton Dohrn, Villa Comunale, 80121 Naples, Italy; mariaconsiglia.esposito@szn.it (M.C.E.); tosti@szn.it (E.T.); 2 Department of Sciences, University of Basilicata, 85100 Potenza, Italy

**Keywords:** ascidian, bovine, fluorescent probe, intracellular reactive oxygen species, mitochondrial activity, mussel, oxidative status, plasma membrane lipid peroxidation, sperm motility, spermatozoon

## Abstract

Sperm quality assessment is the first step for evaluating male fertility and includes the estimation of sperm concentration, motility, and morphology. Nevertheless, other parameters can be assessed providing additional information on the male reproductive potential. This study aimed to evaluate and correlate the oxidative status, mitochondrial functionality, and motility in spermatozoa of two marine invertebrate (*Ciona robusta* and *Mytilus galloprovincialis*) and one mammalian (*Bos taurus*) species. By combining fluorescent staining and spectrofluorometer, sperm oxidative status was evaluated through intracellular reactive oxygen species (ROS) and plasma membrane lipid peroxidation (LPO) analysis. Mitochondrial functionality was assessed through the mitochondrial membrane potential (MMP). In the three examined species, a negative correlation emerged between sperm motility vs ROS levels and LPO. Sperm motility positively correlated with MMP in bovine, whereas these parameters were not related in ascidian or even negatively related in mussel spermatozoa. MMP was negatively related to ROS and LPO levels in ascidians, only to LPO in bovine, and positively related in mussel spermatozoa. These results suggest that energy sources for sperm motility vary between species and that ROS causes a decline in sperm motility via oxidative damage of membrane lipids. Overall, this study validates the use of fluorescent probes in combination with spectrofluorometer as a simple and powerful methodology for supplementary evaluation of sperm quality shedding light on new potential quality markers and provided relevant information on sperm energetic metabolism.

## 1. Introduction

The spermatozoon is a highly specialized haploid cell generated in the male gonad through a differentiation process called spermatogenesis. The function of spermatozoon is to deliver the paternal genome into the female gamete during fertilization, the very special event in which the two gametes fuse their genomes originating a zygote, the first cell of a new diploid individual. To accomplish this task, spermatozoa are equipped with specific structures: a flagellum, which generates the movement, and a very compact nucleus that ensures protection of the paternal genome.

Sperm motility is an essential requirement to ensure the fertilization process and its activation is induced by ionic changes or compounds released from the oocyte or female reproductive tract and implicates the activation of both the motility apparatus and energy metabolism. The motility apparatus is localized into the flagellum and consists of a highly organized microtubule-based structure called axoneme; this organization is well conserved through evolution [1]. Flagellar movement requires an adequate supply of energy in the form of ATP, which is used by the flagellar dynein-ATPase localized into the axoneme along the entire length of the flagellum [2]. A relevant source of ATP necessary to sustain flagellar movement is mitochondrial respiration. Mitochondria are located below the sperm head or in the proximal part of the flagellum tightly wrapped around the axoneme and produce ATP via oxidative phosphorylation. Several studies report that proper mitochondrial functionality is required for ensuring a high sperm quality [3,4]. Structural and functional mitochondrial alterations have been related to loss of sperm function, in particular motility [5], confirming the important role played by these organelles in sperm quality [6]. Besides oxidative phosphorylation, glycolysis is another well-described metabolic pathway that supplies energy for sperm motility; this is an anaerobic process, which takes place in the head and the principal piece of the flagellum [7].

Besides their basic role in ATP synthesis, mitochondria give rise to reactive oxygen species (ROS) as by-products of their activity. ROS is a large class of molecules that includes radicals (hydroxyl ion, superoxide, nitric oxide, peroxyl, etc.), non-radicals (ozone, single oxygen, lipid peroxides, hydrogen peroxide), and oxygen derivatives. At physiological concentrations, ROS activated intracellular pathways underling sperm maturation, capacitation, hyperactivation, acrosome reaction, and gamete fusion [8]. However, when ROS production overwhelms the cellular antioxidant defense systems, a state of oxidative stress occurs. The spermatozoa are particularly susceptible to oxidative stress due to their low antioxidative capacity [9]. Furthermore, the plasma membrane of spermatozoa is characterized by large amounts of polyunsaturated fatty acids highly susceptible to oxidation. Plasma membrane lipid peroxidation (LPO) is highly detrimental to sperm function causing a decrease in membrane fluidity, which in turn affects sperm motility [9]. The assessment of sperm oxidative status and mitochondrial functionality is gaining importance due to their strict correlation with male reproductive function; this brought to develop several methodologies for their evaluation. Sperm mitochondrial function can be investigated through the assessment of mitochondrial membrane potential (MMP), mitochondrial calcium levels, or oxygen consumption [4]. Nowadays, mitochondrial function is commonly assessed by employing fluorescent probes, which accumulate in the mitochondrial matrix owing to the proton motive force after crossing cell membrane and cytoplasm [10]. Currently, different fluorescent potentiometric dyes are used to assess MMP in spermatozoa, such as DiOC6 (3,3′-dihexyloxacarbocyanine iodide), Rh123 (rhodamine-123), tetramethylrhodamine methyl (TMRE), several mito trackers, and JC-1 (5,5,6,6-tetrachloro-1,1,3,3-tetraethylbenzimidazolylcarbocyanine iodide). Among these, the carbocyanine fluorescent dye JC-1 specifically accumulates within mitochondria allowing the most accurate measurement of MMP changes in somatic as well as in sperm cells [11,12,13,14,15,16,17,18]. Differently from rhodamines and other carbocyanines, JC-1 is a ratiometric probe producing two emission peaks, which correspond to two different forms of the dye, monomer, and aggregate. The JC-1 monomers are predominant in depolarized mitochondria characterized by a low MMP and emit green fluorescence, whereas the JC-1 aggregates accumulate in hyperpolarized mitochondria with a high MMP and emit red fluorescence. Upon decreasing MMP, the JC-1 aggregates dissipate into monomers causing a shift from red to green fluorescence.

Nowadays, a close relationship between high levels of intracellular ROS and male reproductive dysfunctions have been demonstrated promoting the development of different methods of ROS detection and quantification. Among these, fluorescent probes have been demonstrated to provide high specificity, accuracy, sensitivity, and reproducibility for intracellular ROS detection [19]. Several redox-sensitive fluorescent probes are commercially available, which can be loaded into spermatozoa and then assessed by fluorescent microscopy, flow cytometry, or spectrofluorimetry. Based on the nature and objective of the subsequent assessment, different fluorescent probes may be used; in particular, cellular H_2_O_2_ production can be detected by the 2,7-dichlorofluoresceindiacetate (H_2_DCFDA), while dihydroethidium (DHE) can be employed to measure cytosolic superoxide anion (O_2_^−•^). H_2_DCFDA is a stable, nonfluorescent cell-permeable probe that undergoes de-esterification in the presence of intracellular H_2_O_2_ to form a fluorescent 2,7-dichlorofluorescein (DCF) [19]. DHE is a hydrophobic uncharged compound, which can penetrate cells freely and is oxidized by the free intracellular O_2_^-^ forming two fluorescent products, the unspecific oxidation product ethidium (Etd), and the highly specific ROS indicator 2-hydroxyethidium (2HE). The emission spectra of these two products overlap; however, the 2HE spectrum can be selectively detected [20].

LPO is considered a specific indicator of oxidative stress in sperm cells and, primarily, it has been assessed detecting the end products of lipid peroxidation such as malondialdehyde. This method is highly sensitive; however, it is complex and time-consuming. Moreover, it does not allow the evaluation of living spermatozoa in real time. As an alternative, the lipophilic fluorescent probe, 4,4-difluoro-5-(4-phenyl-1,3-butadienyl)-4-bora-3a,4adiaza-s-indacene-3-undecanoic acid (C_11_-BODIPY^581/591^), has been widely used to assess LPO in sperm cells. This probe shifts from red to green emission upon oxidation and the ratio of red and green fluorescence has been used as a measurement of LPO (Pap et al., 2000). The fluorescence changes in C_11_-BODIPY^581/591^ reflect indirectly the oxidation of unsaturated fatty acids [21], and the peroxidation rate of C_11_-BODIPY^581/591^ is comparable to that of endogenous fatty acids. Furthermore, this probe has the advantage of being used in a ratiometric method reducing problems with artifacts due to differences in uptake and distribution [22].

Sperm motility, MMP, and oxidative status are three parameters commonly assessed to estimate sperm quality [23,24,25]. Nonetheless, studies concerning the simultaneous evaluation of these parameters are still scarce [26,27] and, in particular, those aimed to correlate these parameters with each other [3,4,16,28,29].

This study aimed to simultaneously evaluate the correlation between the oxidative status, mitochondrial activity, and motility in spermatozoa of two marine invertebrate (the ascidian *Ciona robusta* and the mussel *Mytilus galloprovincialis*) and one mammalian (*Bos taurus*) species. By using fluorescent staining coupled with spectrofluorimetric analysis, the oxidative status was evaluated through the analysis of intracellular ROS levels (O_2_^−•^ and H_2_O_2_) and LPO, whereas mitochondrial functionality was assessed by measuring the MMP. Associations between these parameters may provide valuable information on sperm metabolism and shed light on new potential markers for sperm quality assessment.

## 2. Materials and Methods

All chemicals and dyes were purchased from Merck (Milan, Italy) and Life Technologies (Milan, Italy), respectively.

### 2.1. Sperm Collection and Preparation

#### 2.1.1. Bovine

Frozen/thawed bovine semen samples from 21 Holstein Friesian bulls of proven fertility were used. For each bull, one straw was thawed for 10 sec in the air and then immersed in a warm water bath (37 °C for 30 sec). Three straws were processed in each experiment (*n* = 7). Spermatozoa were layered over 45% and 80% percoll gradient and centrifuged for 30 min at 200× *g*. After washing in a TRIS-fructose medium (247.7 mM Tris (hydroxymethyl) aminomethane, 57.8 mM citric acid, 69.4 mM fructose, 3 mg bovine serum albumin/mL and 1× Penicillin-Streptomycin solution; pH = 7.5, 291 m Osm) and centrifugation at 200 g for 10 min, the pellet was resuspended in TRIS-fructose medium and sperm concentration and motility was assessed by using a Makler counting chamber.

#### 2.1.2. Mussels

The collection of the mussels *Mytilus galloprovincialis* was performed during the breeding season in the bay of Naples. Animals were transported in a cooler bag to the Marine Animal Conservation and Public Engagement (CAPE) department (Stazione Zoologica Anton Dohrn), where their sexual maturity was assessed by sacrificing some animals and evaluating morphological and seminal characteristics. Afterwards, a sorting of the ripe mussels was performed to remove small, dead, and deformed animals and those with shell damage. The selected mussels were acclimated in glass tanks filled with circulating natural seawater (NSW, 38 g/L salinity, pH 8.2 ± 0.1, 16 °C) for six days. Spermatozoa were collected from four males each experimental day (*n* = 5) by opening mussels with a scalpel, cutting the adductor muscle, and then their mantles; finally, the resulting sperm mass was harvested dry with a Pasteur pipette. After collection, sperm suspension was passed through a 50 mm nylon mesh and centrifuged for 15 min at 800× *g* and 4 °C. Then, spermatozoa were suspended in 1 mL of filtered NSW (FNSW, Millipore 0.22 mm; MilliQ, Medford, MA) and maintained at 4 °C. Concentration and motility of spermatozoa were evaluated using the Makler chamber. Finally, the desired sperm concentration was prepared by adding FNSW.

#### 2.1.3. Ascidians

Adult individuals of *Ciona robusta* were sampled in the Gulf of Naples (Italy), transported into a cool box to the CAPE department, where animals were acclimated in glass thanks filled with circulating seawater at 18 °C for at least two days until the experiments. A total of 21 animals were used across five experiments. After anesthetization on ice, spermatozoa were collected dry with a Pasteur pipette from the sperm ducts, and concentration and motility were assessed using the Makler chamber. Finally, the desired sperm concentration was prepared by adding FNSW.

#### 2.1.4. Ethics Approval

This study has been conducted according to the guidelines of the Declaration of Helsinki and amended by the European Directive 2010/63 on the protection of animals used for scientific purposes, transposed into the Italian law by Legislative Decree 2014/26. 

### 2.2. Sperm Motility

In bovine, sperm motility was evaluated using the Sperm Class Analyzer (SCA) 5.0 system (Microptic, Barcelona, Spain). A sperm suspension at the final concentration of 20 × 10^6^ spermatozoa/mL was prepared by adjusting sperm concentration with TRIS-fructose medium. Then, an aliquot of sperm suspension was loaded into the Makler chamber and examined using a Labophot microscope (Nikon, Tokyo, Japan) with a warmed stage (37 °C) and equipped with a 10x Bright Medium negative phase contrast objective. Sperm motility patterns were recorded using a monochrome video camera (CCD Hitachi, Tokyo, Japan) and analyzed with the SCA system operating at 25 video frames per sec (25 Hz). The search radius was 11.49 µm, and spermatozoa with an average velocity of less than 10 µm/sec were considered immotile.

In mussels and ascidians, sperm motility was evaluated with the Computer-Assisted Sperm Analyzer (CASA) application of the open-source ImageJ software, as previously reported [13]. Briefly, a sperm aliquot (50 × 10^6^ spermatozoa/mL) was loaded into the Makler chamber and sperm movements were registered for 2 sec at 50 frames/sec with a digital camera (Axiocam 506mono, Ziess, Germany) mounted on a microscope (Axio Imager Z.2, Zeiss, Germany) equipped with a 10X A-Plan phase contrast objective. The acquired videos were analyzed by CASA [30]. For each semen sample, three microscopic fields were randomly assessed and the percentage of motile spermatozoa was calculated as the mean measure of three scans for each microscopic field.

### 2.3. Mitochondrial Membrane Potential Assessment

The MMP was evaluated with the JC-1 vital mitochondrial dye, which reversibly changes from aggregate to monomer forms upon variation in MMP shifting the emitted fluorescence from red (~595 nm) to green (~535 nm).

Sperm aliquots at concentration of 1 × 10^6^ spermatozoa/mL were incubated in 5 µM JC-1 for 30 min in the dark at 37 °C for bovine or at 18 °C for ascidian and mussel and then centrifuged at 200× *g* for 10 min at 37 °C (bovine) or 800× *g* for 10 min at 4 °C (ascidian and mussel). Finally, the pellets were suspended in the specific medium and transferred to quartz cuvette (10 × 4 mm, high precision cell, Hellma Analytics, Müllheim, Germany) for spectrofluorometric analysis. The fluorescence spectra were recorded in duplicate for each sample setting the excitation wavelength at 488 nm and recording the emission spectrum in the range of 500–650 nm. The MMP was calculated as a ratio of the fluorescence peak values at ~595 nm (F_0_B) and ~535 nm (F_0_A). For the positive control, an aliquot of JC-1-loaded spermatozoa was incubated with 5 µM of the protonophore mitochondrial uncoupler carbonyl cyanide m-chlorophenyl hydrazone (CCCP).

### 2.4. Intracellular ROS levels

Intracellular H_2_O_2_ levels were assessed with the 2′,7′-dichlorodihydrofluorescein diacetate (H_2_DCFDA). Sperm aliquots diluted at 1 (bovine) or 5 (ascidians and mussels) × 10^6^ cells/mL were stained with 10 µM H_2_DCFDA for 30 min in the dark at 37 °C for bovine or 18 °C for ascidian and mussel, washed and further incubated for 30 min in their specific medium. For the positive control, an aliquot of sperm suspension was incubated with 2.5 µM hydrogen peroxide. After incubation, samples were centrifuged, pellets suspended in the specific medium, and then transferred to quartz cuvette for spectrofluorometric analysis. The peak fluorescence intensity (peak at ~525 nm) of emission spectrum recorded from 500 to 560 nm wavelengths with an excitation wavelength of 488 nm is proportional to the intracellular H_2_O_2_ levels (Arbitrary Units, A.U.).

The intracellular content of O_2_^-^ was assessed by employing the fluorophore DHE. Aliquots of spermatozoa, 3 (bovine) and 30 (ascidians and mussels) × 10^6^ spermatozoa/mL, were incubated with 20 µM DHE for 30 min in the dark at 37 °C (bovine) or 18 °C (ascidians and mussels). After incubation, samples were transferred to a spectrofluorometric cuvette. For the positive control, an aliquot of sperm suspension was incubated with 20 µM of the redox-active quinone menadione, which induces superoxide anion formation. Fluorescence intensity peaks (Arbitrary Units, A.U.) were recorded setting an excitation wavelength of 350 nm and the emission wavelengths from 550 to 650 nm.

### 2.5. Lipid Peroxidation Assessment

LPO was detected with the fluorescent fatty acid analog dye C11-BODIPY^581/591^, which easily inserts into the lipid bilayer of membranes and, upon oxidation, its emission spectrum shifting from red (~595 nm) to green (~520 nm) fluorescence. Sperm suspensions diluted at 1 (bovine) or 5 (ascidians and mussels) × 10^6^ cell/mL were incubated with 2 μM C11-BODIPY^581/591^ for 30 min in the dark at 37 °C (bovine) or 18 °C (ascidians and mussels). After staining, samples were centrifuged, the pellets suspended in the specific medium and transferred to a spectrofluorometric cuvette. For the positive control, an aliquot of sperm suspension was incubated with two peroxidation promoters, i.e., 150 μM ferrous sulfate and 750 μM ascorbic acid. The fluorescence intensity measurement was performed setting the excitation wavelength at 488 nm and emission wavelengths from 500 to 650 nm. Finally, to perform a ratiometric analysis, the fluorescence emission peak value recorded at ~520 nm (F_0_A) was related to the sum of fluorescence emission peak values at ~520 (F_0_A) and ~590 nm (F_0_B).

### 2.6. Data Analysis

A dataset containing data from ascidian, mussel, and bovine sperm samples was organized as follows: the experimental data, the sample and replication number, total sperm motility (Tot Mot), MMP, LPO, H_2_O_2_, and O_2_^-^ values.

To assess the normal data distribution and the variance homogeneity assumption that are required to perform a parametric test, the Shapiro–Wilks test and the Levene’s test were used, respectively. The linear regression procedure (Systat 11.0 release) was applied to calculate the coefficients of correlation (R). A *p*-value ≤ 0.05 was set as the minimum level of statistical significance. Data are reported as mean ± standard deviation (SD).

## 3. Results

### 3.1. Data Report

The mean values (±SD) of total motility, MMP, LPO, intracellular content of H_2_O_2_ and O_2_^−^ recorded in the mammalian (bovine), and marine invertebrate (ascidians and mussels) species are reported in Table 1. Without statistically comparing such different species, however, it is noted that sperm motility is higher in the frozen bovine than fresh spermatozoa of the two marine invertebrates. This may be attributed to the selection of the sperm quality of the bovine semen before its freezing. This selection was not carried out for the marine species examined. MMP is lower in bovine spermatozoa, while LPO appears higher in ascidian spermatozoa. The ROS content is also substantially lower in cattle than in the two marine invertebrates.

### 3.2. Correlation Analysis

Data obtained from the simultaneous assessment of sperm motility, MMP, LPO as well as the intracellular levels of H_2_O_2_ and O_2_^-^ in the three examined species were analyzed by linear regression procedure and the relative correlation coefficients were reported in Table 2 (Correlation plots are included in supplementary materials; Figures S1–S3). In bovine spermatozoa, motility was positively correlated with MMP (r= +0.683; *p* = 0.001) and negatively correlated with LPO (r = −0.890; *p* = 0.001) and H_2_O_2_ levels (r = −0.742; *p* = 0.001). MMP was negatively correlated to LPO (r = −0.621; *p* = 0.003). LPO was positively correlated to H_2_O_2_ levels (r = +0.721; *p* = 0.001) but not significantly related to O_2_^−^ levels (r = −0.253; *p* = 0.269).

In ascidian spermatozoa, motility was negatively correlated with LPO (r = −0.660; *p* = 0.001), H_2_O_2_ levels (r = −0.790; *p* = 0.001), and O_2_^−^ levels (r = −0.693; *p* = 0.001); however, it was not significantly correlated with MMP. On the other hand, MMP was negatively correlated with LPO (r = −0.720; *p* = 0.001), H_2_O_2_ (r = −0.701; *p* = 0.001) and O_2_^−^ (r = −0.713; *p* = 0.001). LPO was positively correlated with H_2_O_2_ levels (r = +0.840; *p* = 0.001) and to O_2_^−^ levels (r = +0.946; *p* = 0.001). A significant correlation was also observed between H_2_O_2_ and O_2_^−^ levels (r = +0.830; *p* = 0.001)

In mussel spermatozoa, motility was negatively correlated to MMP (r = −0.637; *p* = 0.001), LPO (r = −0.595; *p* = 0.003), H_2_O_2_ levels (r = −0.650; *p* = 0.001) and O_2_^−^ levels (r = −0.608; *p* = 0.003). MMP was positively correlated to LPO (r = +0.483; *p* = 0.020), H_2_O_2_ levels (r = +0.605; *p* = 0.003), and O_2_^−^ levels (r = +0.775; *p* = 0.001). Moreover, H_2_O_2_ levels was positively related to O_2_^−^ levels (r = +0.649; *p* = 0.002).

## 4. Discussion

This study investigates for the first time the correlation between the oxidative status, mitochondrial activity, and motility in bovine and two marine invertebrate (the ascidian *C. robusta* and the Mediterranean mussels *M. galloprovincialis*) spermatozoa. This correlation analysis shed a light on new potential markers, which could be introduced in the conventional assessment of sperm quality.

Attempts to identify new predictors of sperm quality have focused on mitochondrial markers. MMP closely reflects mitochondrial function, so its assessment allows for predicting sperm mitochondrial function and performing a complete evaluation of sperm quality. Indeed, this parameter has been demonstrated to be associated with functional sperm parameters [16].

Nowadays, a close relationship between mitochondria functionality and sperm motility is widely demonstrated in humans; unfortunately, it has not yet been explored in many other mammalian species and even less in invertebrates. In particular, a high positive correlation between sperm motility and mitochondrial activity has been demonstrated in humans, horses, rodents, and boars, suggesting that, in these species, motility relies almost entirely on oxidative phosphorylation for the ATP generation [3,15,16,31,32]. Here, in accordance with a previous study on canine spermatozoa [33], a moderate correlation between motility and mitochondrial activity was found in bovine spermatozoa suggesting that, in this species, sperm motility does not entirely rely on oxidative phosphorylation, but it is likely a cooperation between glycolysis and oxidative phosphorylation for the ATP generation.

In marine invertebrates, motility and mitochondrial activity were widely investigated in spermatozoa of several species after exposure to diverse physical and chemical stressors [34,35,36,37,38,39,40]; however, based on our knowledge, a correlation between these parameters has never been performed. Our results reveal that total motility and MMP are not associated (*C. robusta*) or even negatively related (*M. galloprovincialis*) suggesting that, in these species, oxidative phosphorylation may not represent the major source of energy for sperm motility.

Compared to glycolysis, oxidative phosphorylation is more efficient in ATP generation; however, during oxidative phosphorylation, a leakage of electrons over the electron transport chain normally occurs resulting in ROS production. The excessive generation of ROS may be extremely harmful to spermatozoa, which are deficient in antioxidant defenses and sourceof oxidative stress. Many reports correlate oxidative stress with sperm function impairment; in particular, a negative correlation between intracellular ROS levels and sperm morphology, concentration, and motility has been widely demonstrated [41,42,43]. Nevertheless, recently, the widely accepted dogma that ROS is a negative indicator of sperm functionality has been questioned [4,9]. Indeed, some studies have demonstrated the absence of a significant relationship between intracellular ROS levels and sperm motility [44,45]; on the contrary, in equine spermatozoa, a positive correlation between ROS production and motility has been demonstrated [46]. In this study, in bovine and ascidian spermatozoa, the intracellular ROS levels negatively correlated to motility and MMP, and positively are related to LPO. These results suggest that an increase in intracellular ROS levels may induce mitochondrial dysfunction and a decline in sperm motility via oxidative damage of membrane lipids. In particular, the high production of ROS induces the lipid peroxidation of both cellular and mitochondrial plasma membrane altering the fluidity and the integrity of these structures. These, in turn, cause a decrease in sperm motility and the upregulation of proton and electron leakage through the inner mitochondrial membrane leading to the loss of MMP, which affects mitochondrial ATP generation efficiency and promotes mitochondrial ROS generation.

In mussel spermatozoa, motility was also negatively related to intracellular ROS levels and LPO; furthermore, increased levels of ROS and LPO have been correlated with the increase of MMP. All together, these results suggest that, in metabolically active spermatozoa, the intracellular ROS levels increase leading to LPO, and, consequently, to a decrease in sperm motility.

LPO is a condition resulting from the balance between oxidative stimuli and intra- and extracellular antioxidant defenses. Our results indicate that H_2_O_2_ species may be the main inducer of LPO in bovine spermatozoa, whereas, in ascidian spermatozoa, it is induced by both H_2_O_2_ and O_2_^−^ species, which, on the contrary, are not related to LPO in mussel spermatozoa. To date, the etiology of oxidative stress in spermatozoa, in particular in marine invertebrates, has not yet been elucidated; therefore, the different relationships observed between ROS and LPO in the three examined species cannot be clearly explained. It may be due to the dynamics of LPO occurrence or the generation of different reactive species under diverse metabolic conditions of these gametes. In human spermatozoa, it has been demonstrated that ROS production is not correlated with LPO under standard culture conditions following short incubation times [47]. However, a positive correlation was observed after the induction of oxidative stress in human sperm exposure to iron salts. Hence, LPO occurs under normal conditions with different timing than that related to ROS production. Studies in bovine spermatozoa supported this pattern [12].

Overall, this study indicates that the metabolic pathway opted by spermatozoa for energy production is highly species-specific and that oxidative phosphorylation may play a minor role in the energy metabolism of marine invertebrate spermatozoa. Moreover, it suggests that LPO may be a preserved pathway by which the high production of ROS leads to decreased sperm motility. Indeed, MMP, ROS, and LPO may be considered new potential markers of sperm quality, which provide additional information on sperm reproductive potential.

## 5. Conclusions

This study validates a new approach to investigate different sperm quality parameters (MMP, ROS, and LPO) by combining fluorescent staining and spectrofluorometric and correlate these parameters with each other to evaluate their possible use as new predictors of sperm quality. Our results reveal different relationships between sperm motility and mitochondrial activity in the three examined species suggesting that the energy sources for sperm motility vary between species.

On the contrary, in all examined species, sperm motility negatively correlates with intracellular ROS levels and LPO, whereas ROS and LPO are positively correlated. All together, these results suggest that the high intracellular ROS levels in spermatozoa may be a cause of male infertility resulting in a loss of sperm motility via oxidative damage of membrane lipids in vertebrate as well as invertebrate species. 

Hence, MMP, intracellular ROS levels, and LPO may be quick, cost-effective, and reliable markers of sperm quality for use in the conventional sperm quality assessment. Moreover, the assessment of these parameters may provide valuable information for the development of treatment strategies aimed at improving the male’s reproductive potential.

## Figures and Tables

**Table 1:** Mean (±SD) values of total motility (Tot Mot), mitochondrial membrane potential (MMP) evaluated by JC-1, lipid peroxidation (LPO) evaluated by C11-BODIPY^581/591^, intracellular content of hydrogen peroxide (H_2_O_2_) evaluated by 2′,7′-dichlorodihydrofluorescein diacetate (H_2_DCFDA) and of superoxide anions (O_2_^−^) evaluated by dihydroethidium (DHE) in spermatozoa of the three species examined.

Species	Tot Mot (%)	MMP (F_0_B/F_0_A)	LPO (F_0_A/(F_0_A + F_0_B)) × 100	H_2_O_2_ (A.U.)	O_2_^−^ (A.U.)
*Bos taurus*	58.2 ± 26.3	4.2 ± 3.7	28.0 ± 17.1	26.5 ± 10.8	32.6 ± 13.8
*Ciona robusta*	42.2 ± 16.5	8.8 ± 6.6	37.7 ± 3.4	159.8 ± 46.8	97.6 ± 10.8
*Mytilus galloprovincialis*	42.9 ± 27.5	11.5 ± 6.1	28.2 ± 9.7	607.5 ± 478.7	247.6 ± 98.0

F_0_A and F_0_B: fluorescence intensity peaks; A.U.: international units.

**Table 2:** Correlation coefficients (R) between sperm total motility (Tot Mot), mitochondrial membrane potential (MMP), lipid peroxidation (LPO), intracellular content of hydrogen peroxide (H_2_O_2_) and of superoxide anions (O_2_^−^**)** in the three species examined.

Species		MMP	LPO	H_2_O_2_	O_2_^−^
*Bos taurus*	Tot Mot MMP LPO H_2_O_2_	+0.683 ***	−0.890 *** −0.621 **	−0.742 *** −0.320 +0.721 ***	+0.299 +0.334 −0.253 −0.398
*Ciona robusta*	Tot Mot MMP LPO H_2_O_2_	+0.272	−0.660 * −0.720 ***	−0.790 *** −0.701 *** +0.840 ***	−0.693 *** −0.713 *** +0.946 *** +0.830 ***
*Mytilus galloprovincialis*	Tot Mot MMP LPO H_2_O_2_	−0.637**	−0.595 ** +0.483 *	−0.650 ** +0.605 ** +0.288	−0.608 ** +0.775 ** +0.208 +0.649 **

* (*p* ≤ 0.05), ** (*p* ≤ 0.01), *** (*p* ≤ 0.001).

## Data Availability

Data is contained within the article and supplementary materials.

## Supplementary Materials

The following are available online at https://www.mdpi.com/article/10.3390/antiox10071131/s1, Figure S1: Correlation plots between Total motility (Tot Mot), mitochondrial membrane potential (MMP), Lipid peroxidation (LPO), H2O2 and O2- contents in Bos taurus spermatozoa, Figure S2: Correlation plots between Total motility (Tot Mot), mitochondrial membrane potential (MMP), Lipid perox-idation (LPO), H2O2 and O2- contents in Ciona Robusta spermatozoa, Figure S3: Correlation plots between Total motility (Tot Mot), mitochondrial membrane potential (MMP), Lipid peroxidation (LPO), H_2_O_2_ and O_2_^-^ contents in *Mytilus galloprovincialis* spermatozoa.
