# Supplementary material for: Sperm Motility, Oxidative Status, and Mitochondrial Activity: Exploring Correlation in Different Species
Source: Antioxidants (Basel). 2021 Jul 16;10(7):1131. doi: 10.3390/antiox10071131 (PMC8301117; doi:10.3390/antiox10071131)
Supplement: Supplementary file 1 [file antioxidants-10-01131-s001.zip › antioxidants-1289021-supplementary.pdf]

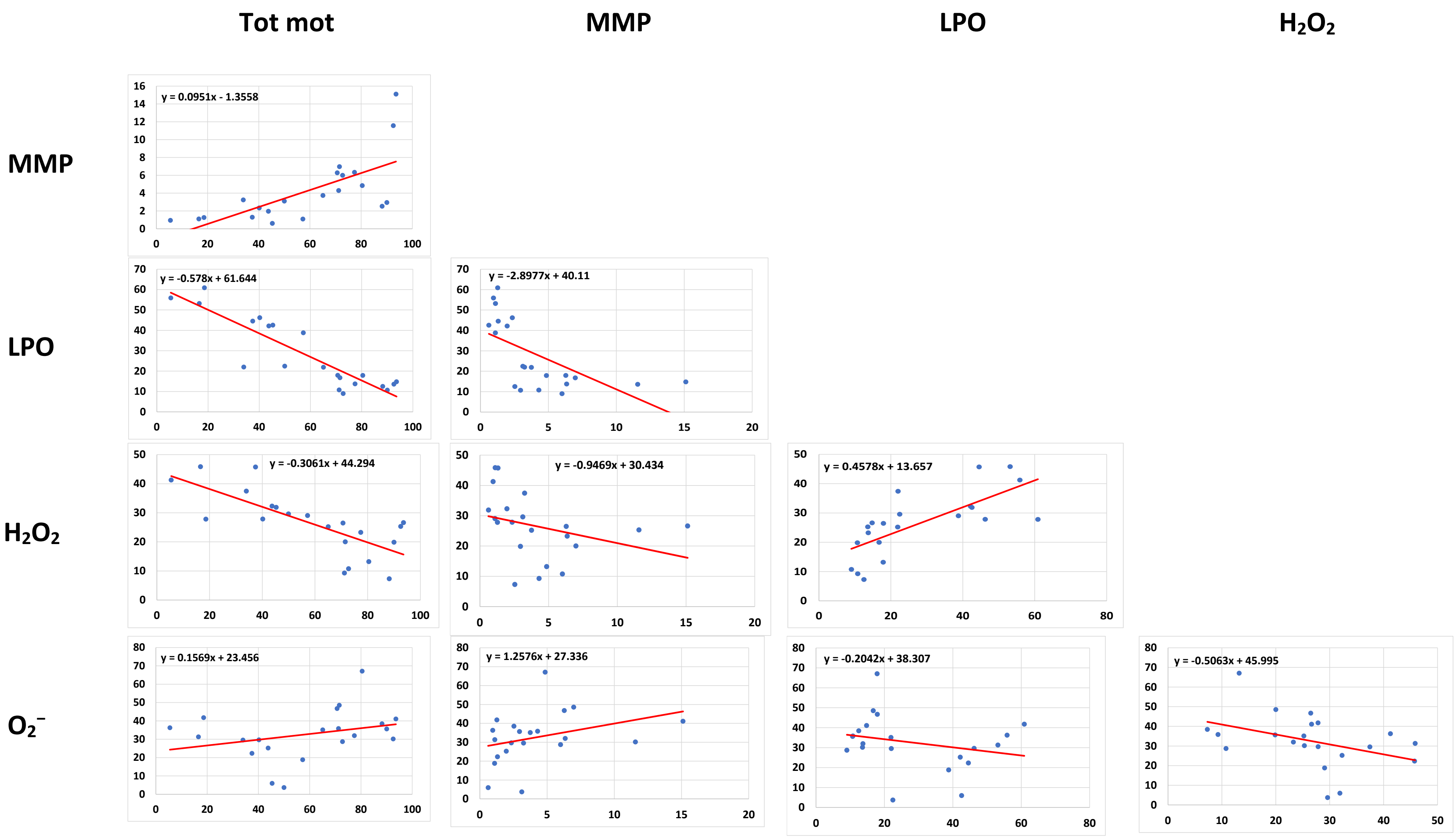

Figure S1. Correlation plots between Total motility (Tot Mot), mitochondrial membrane potential (MMP), Lipid peroxidation (LPO), H_2O_2 and O_2^- contents in *Bos taurus* spermatozoa.

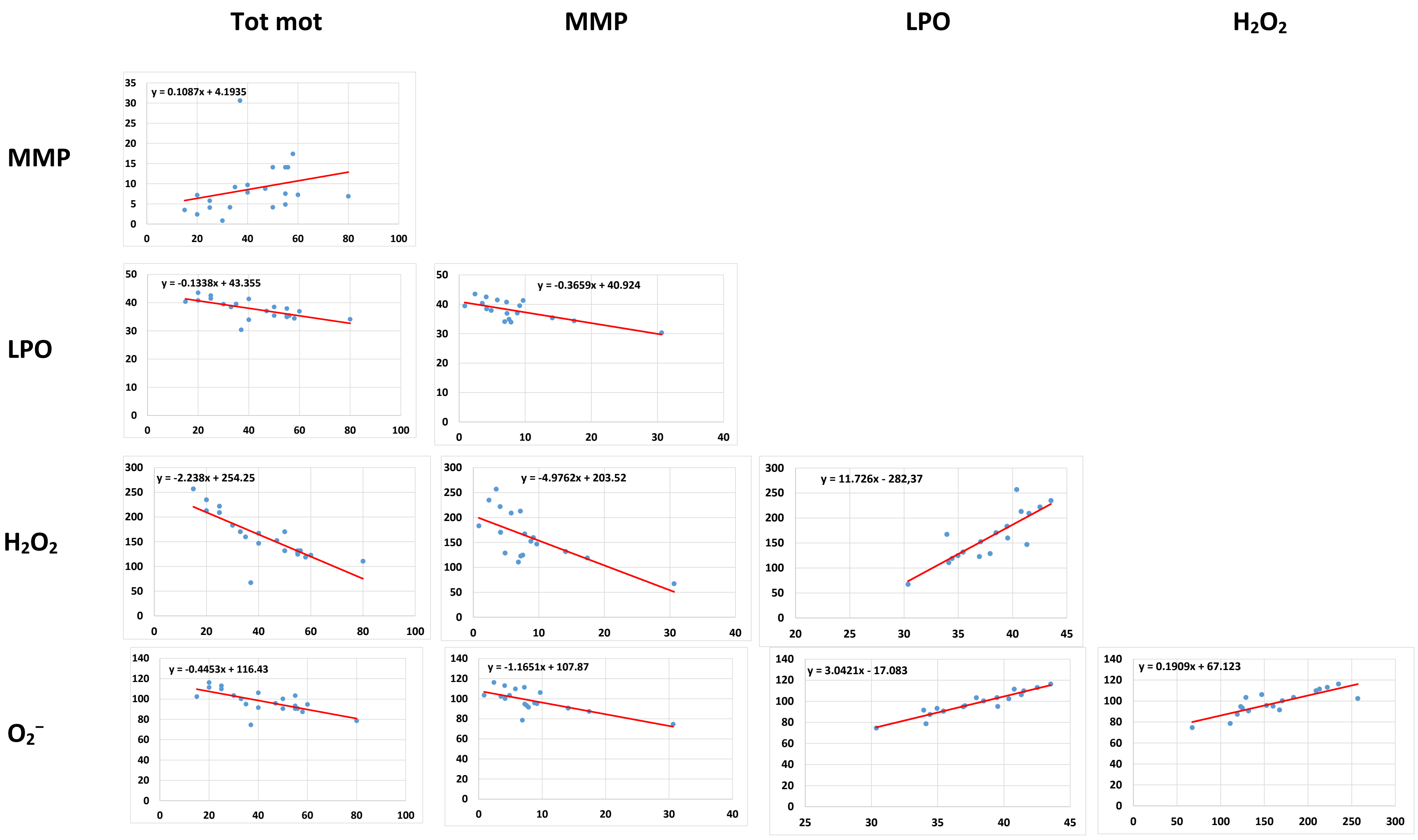

Figure S2. Correlation plots between Total motility (Tot Mot), mitochondrial membrane potential (MMP), Lipid peroxidation (LPO), H_2O_2 and O_2^- contents in *Ciona Robusta* spermatozoa.

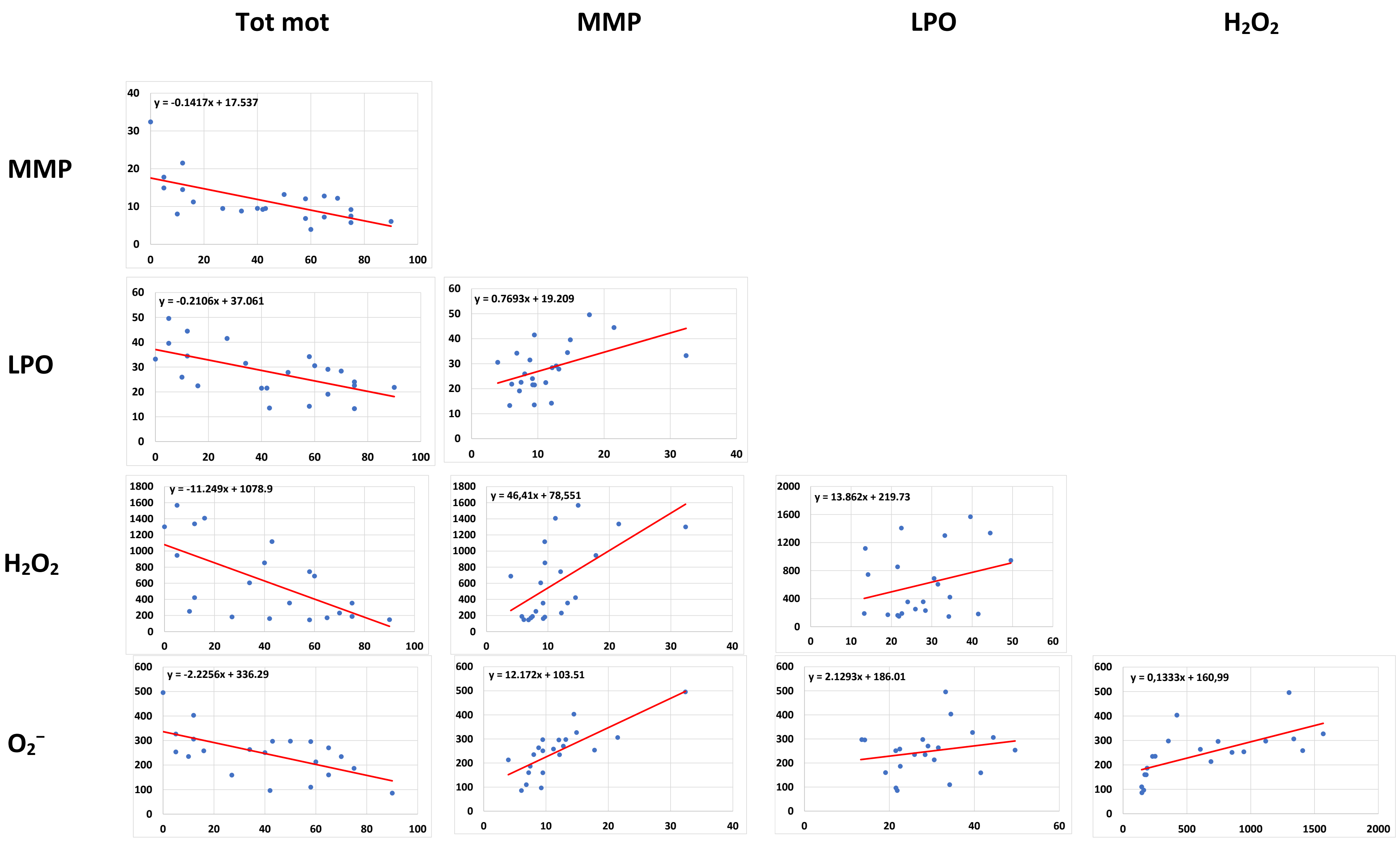

Figure S3. Correlation plots between Total motility (Tot Mot), mitochondrial membrane potential (MMP), Lipid peroxidation (LPO), H_2O_2 and O_2^- contents in *Mytilus galloprovincialis* spermatozoa.
